# Deciphering collaborative sidechain motions in proteins during molecular dynamics simulations

**DOI:** 10.1038/s41598-020-72766-1

**Published:** 2020-09-28

**Authors:** Bruck Taddese, Antoine Garnier, Hervé Abdi, Daniel Henrion, Marie Chabbert

**Affiliations:** 1 CNRS UMR 6015 – INSERM U1083, MITOVASC Laboratory, University of Angers, Angers, France; 2 School of Behavioral and Brain Sciences, The University of Texas at Dallas, Dallas, USA

**Keywords:** Biophysics, Computational biology and bioinformatics

## Abstract

The dynamic structure of proteins is essential for their functions and may include large conformational transitions which can be studied by molecular dynamics (MD) simulations. However, details of these transitions are difficult to automatically track. To facilitate their analysis, we developed two scores of correlation between sidechain dihedral angles. The *CIRCULAR* and *OMES* scores are computed from, respectively, dihedral angle values and rotamer distributions. As a case study, we applied our methods to an activation-like transition of the chemokine receptor CXCR4, observed during accelerated MD simulations. The principal component analysis of the correlation matrices was consistent with the networking structure of the top ranking pairs. Both scores identify a set of residues whose “collaborative” sidechain rotamerization immediately preceded or accompanied the conformational transition of CXCR4. Detailed analysis of the sequential order of these rotamerizations suggests that an allosteric mechanism, involving the outward motion of an asparagine residue in transmembrane helix 3, might be a prerequisite to the large scale conformational transition of CXCR4. This case study provides the proof-of-concept that the correlation methods developed here are valuable exploratory techniques to help decipher complex reactional pathways.

## Introduction

With more than 150,000 protein structures deposited in the Protein Data Bank (PDB) by the end of 2019, structural biology has provided breakthrough advances in the functional mechanisms of numerous proteins and in drug design^1^. The crystallographic structures deposited in the PDB give examples of the large amplitude conformational changes that proteins can undergo under specific conditions. However, the crystal structures provide static snapshots of protein structure in a specific conformation or in different conformations but do not provide details about their intrinsic mobility or into the molecular mechanisms of conformational changes.

Proteins are inherently dynamic structures that undergo motions on a large range of both timescales and amplitude^2^. Local motions include bond vibrations on the femtosecond timescale, sidechain rotamerization on the picosecond to the microsecond timescale, loop motions on the nanosecond to the microsecond timescale. Collective motions have larger amplitude and lead to larger domain motions that may occur on the microsecond to second or larger timescale. The experimental techniques developed to access these motions can be complemented by molecular dynamics (MD) simulations that provide a dynamic view of protein structures^3–7^.

In recent years, the huge increase in computational power has made it possible to perform MD simulations of hydrated or membrane inserted proteins with computational time up to the millisecond timescale using, for example, the specially-designed supercomputer Anton^8,9^, Google Exacycle^10^ or GPU-based MD simulations^11,12^. However, most classical MD simulations are run on the nanosecond to microsecond timescale. This timescale allows reaching local flexibility but does not provide information on larger domain motions^2^.

To bridge the gap between computationally accessible and biologically relevant timescales, numerous molecular dynamics techniques aimed at accelerating simulations have been developed. These simulations include coarse grained simulations, normal mode analysis and a variety of techniques aimed at reducing the energy barriers between interconverting conformations^4,6^. Among them, accelerated molecular dynamics (aMD) provides boosts of dihedral and potential energies, helping the system to overcome energy barriers^13,14^. The main advantage of aMD is to make no hypothesis on the final conformation that the system can reach.

G protein-coupled receptors (GPCRs) provide an example of large conformational transition upon activation by agonist binding. The crystallographic structures of different GPCRs in inactive and active states have revealed a large outward motion of transmembrane helix 6 (TM6) upon activation. This motion opens the receptor intracellular domain and allows subsequent effector binding^15–18^. The TM6 motion is accompanied by a reorganization of the interactions stabilizing the receptor fold, including side chain rotamerization events to yield stable structures^19–22^. GPCR activation happens on timescales longer than the millisecond and cannot be presently observed by classical MD simulations^23,24^. Observation of deactivation (transition from the “unstable” active structure to the “stable” inactive structure) requires tenths of microseconds of classical simulations^25^, whereas observation of activation is presently possible only with techniques aimed at increased sampling of the conformational space, such as adaptive sampling^12^, biased MD^26^ and accelerated MD^27–29^ simulations.

In this study, we were interested in deciphering the molecular details of a spontaneous activation-like transition of CXCR4, a GPCR from the chemokine receptor family, upon aMD simulations. We had a special concern for highlighting residues that undergo sidechain motion in relation with receptor transition. As sidechain motions are best described in terms of internal coordinates by using dihedral angles^30,31^, we developed an approach based on the correlation of either dihedral angles or rotamer distributions (*CIRCULAR* and *OMES* scores, respectively), and we applied it to our case study. Our approach highlights several sidechains whose quasi-simultaneous rotamerization immediately preceded the conformational transition. Our results suggests that an allosteric mechanism involving the outward motion of an asparagine residue in TM3 (N3.35 in Ballesteros’notation^32^) may facilitate the receptor activation.

## Methods

### Molecular dynamics simulations of CXCR4

In this study, we used a previously described^33^ model of the CXCR4 receptor with a sodium ion bound to the sodium allosteric binding site. Briefly, the model was built with MODELLER by homology with the crystal structure of human CXCR4 in an inactive state (PDB 3ODU)^34^ and with the sodium binding site of the δ opioid receptor (PDB 4N6J)^35^, with reversion of the mutations/insertion present in the crystallized CXCR4 receptor to the amino acids present in the human wild type receptor. Then, the receptor model was embedded in a 1-palmitoyl-2-oleoyl-sn-glycero-3-phosphocholine (POPC) lipid bilayer and hydrated using the CHARMM-GUI interface^36^. The charges were neutralized by adding chloride ions.

The molecular dynamics simulations were carried out with the NAMD v2.9 MD software^37^, using the CHARMM36 forcefield^38,39^, as previously described^33^, except that these simulations were run on the E-biothon cloud platform^40^. The simulations were carried out at 310 K and 1 atm. After a 1 ns equilibration step with progressive release of conformational constraints and increase in step length from 1 to 2 fs^36^, classical MD simulations (cMD) were run for 120 ns. The last coordinates and velocities obtained by cMD were used to start accelerated molecular dynamics simulations lasting for 180 ns. Accelerated MD works by adding non-negative dihedral and potential boosts to the dihedral and potential energies of the system when they are above a threshold. These thresholds were set to, respectively, the average dihedral and potential energies, *E*_dihed_avg_ and *E*_pot_avg_, computed from the cMD trajectory. The acceleration factors were calculated from *E*_dihed_avg_, the average dihedral energy, and *N*, the number of atoms in the system, according to:

1 $$ \alpha_{{{\text{dihed}}}} = \uplambda \times \frac{{E_{{{\text{dihed\_avg}}}} }}{5} $$

2 $$ \alpha_{{{\text{pot}}}} = \lambda \times N $$

where the acceleration parameter *λ* was set to 0.3^27,41^. During both the cMD and aMD simulations, the length of each step was 2 fs. Quantitative analyses were performed with the R package Bio3D^42^. Graphical analysis of the trajectory was carried out with the VMD program^43^. Figures were prepared with the PyMOL Molecular Graphics System, version 1.8 (Schrödinger, LLC). To facilitate comparison with other GPCRs, the position of each residue is followed by a superscript indicating its Ballesteros’ numbering^32^.

### Correlation scores

**Figure 1:**
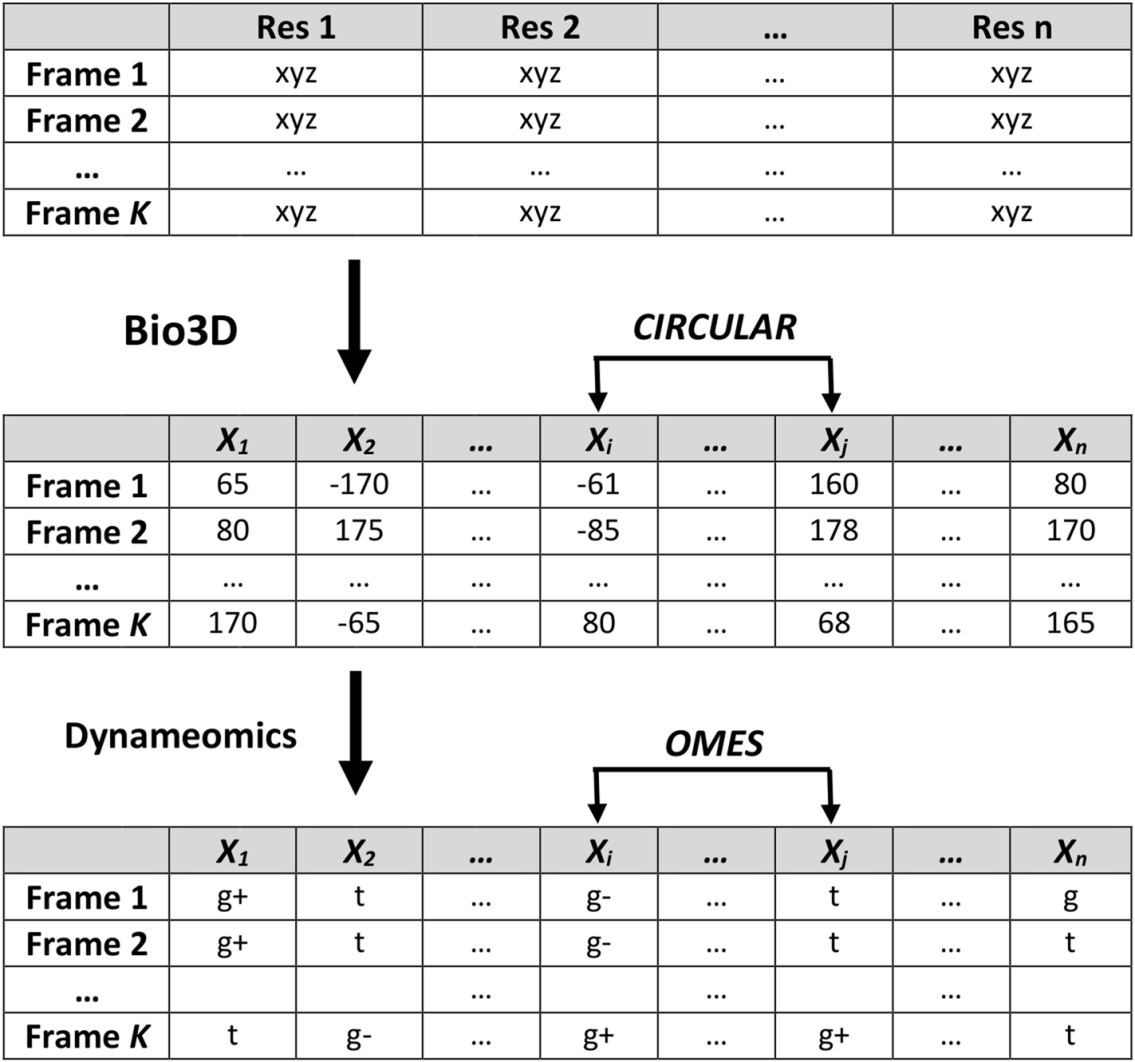
Flow chart used to compute the correlation scores for sidechain dihedral angles. Starting from the Cartesian coordinates of a protein in the *K* frames of a MD trajectory file, the Bio3D package calculates the values of the sidechain dihedral angles at each frame of the trajectory. For each pair (*X*_*i*_, *X*_*j*_) of dihedral angles, *CIRCULAR* measures the correlation between these values. Using the dynameomics library, the dihedral angles are converted into rotamers, then *OMES* measures covariation between rotamers.

The flow chart developed to compute correlation scores between sidechain dihedral angles is displayed in Fig. 1. First, using the R package Bio3D^42^, the Cartesian coordinates of the heavy atoms of the protein were transformed into dihedral angles to build the dihedral matrix which gives the chi dihedral angles of each sidechain (except Gly and Ala) for each frame of the trajectory (488 dihedral angles for 1200 frames in the trajectory under scrutiny). Second, to obtain the rotamer matrix, the values of the dihedral angles were converted into rotamers using the dynameomics library^44^.

We developed two functions to measure correlation scores between two dihedral angles. The first function, *CIRCULAR*, relies on the dihedral angle values (circular continuous variables). It is based on a circular version of the Pearson coefficient^45,46^, that is encoded in the R package circular^47^ and computed as:

3 $$ circular({\text{X}}_{i} \, ,{\text{X}}_{j} ) = \frac{{\sum\limits_{k=1}^{K} {\sin (\alpha_{k} - \overline{\alpha })} \sin (\beta_{k}- \overline{\beta })}}{{\sqrt {\sum\limits_{k=1}^{K} {\sin^{2} (\alpha_{k} - \overline{\alpha })} } \sqrt {\sum\limits_{k=1}^{K} {\sin^{2} (\beta_{k}- \overline{\beta })} } }} $$

where *Χ*_*i*_ and *Χ*_*j*_ are two sidechain dihedral angles, α_*k*_ and β_*k*_ are their angular values at frame *k*, ${\overline{\alpha }}$ and ${\overline{\beta }}$ are the circular averages of α and β, and *K* is the number of frames. Because the sign of the score is irrelevant for our purposes, we used the squared correlation score:

4 $$CIRCULAR({\rm X}_{i} ,{\rm X}_{j} ) = circular({\rm X}_{i} ,{\rm X}_{j} )^{{2}}.$$

The second method relies on rotamer distributions (discrete variables). After conversion of the dihedral angle values into rotamers^44^, the covariation between rotamer occurrences is calculated by the *OMES* method^48^. The *OMES* score of covariation (Observed Minus Expected Squared) was initially developed for the analysis of amino acid covariation in sequence alignments^33,48–50^. The same formalism can be extended to rotamer covariation in MD trajectories. In that case, the *OMES* score for two dihedral angles *Χ*_*i*_ and *Χ*_*j*_ is computed as:

5 $$ OMES({\rm X}_{i} ,{\rm X}_{j} ) = \frac{1}{K}\sum\limits_{x,y} {(N_{x,y}^{{{\text{obs}}}} ({\rm X}_{i} ,{\rm X}_{j} ) - N_{x,y}^{\exp } ({\rm X}_{i} ,{\rm X}_{j} ))^{2} } $$

where *K* is the number of frames, $N_{x,y}^{{{\text{obs}}}} ({\rm X}_{i} ,{\rm X}_{j} )$ and $N_{x,y}^{\exp } ({\rm X}_{i} ,{\rm X}_{j} )$ are the number of frames for which the rotamer pair (*x*, *y*) is observed and expected, respectively, for the dihedral angles *Χ*_*i*_ and *Χ*_*j*_. Expectation is based on the frequency of rotamers *x* and *y* for, respectively, the dihedral angles *Χ*_*i*_ and *Χ*_*j*_.

### Analysis of correlation scores

The *OMES* and *CIRCULAR* scores and the subsequent functions developed for data analysis were written in the R programing language and are available in the R package Bios2cor (version 2.1) which can be found in the Comprehensive R Archive Network (cran.r-project.org).

The correlation/covariation scores between the dihedral angles were stored in squared matrices (here 488 × 448 matrices) whose all diagonal values were set to 0. Scores of correlation/covariation between dihedral angles *within the same sidechain* (autocorrelation) were set to 0 (lowest score for both methods) because we were interested in studying dihedral correlations *between different sidechains*. Thereafter, for clarity purpose, the term “correlation” will be used for both scores.

For comparative purposes, the raw scores were normalized by computing *Z*-scores (i.e., a score is now represented by its number of standard deviations to the mean^51^). This normalization effectively removes the scale of measurement without making any hypothesis about the shapes of the distributions.

We performed two types of analysis. First, we focused on the top ranking pairs and we analyzed their networking structure. The visualization of the network between top ranking pairs of dihedral angles was carried out with the Cytoscape software (version 3.7)^52^. This analysis was limited to the top 25 pairs—a number that represents about 5% of the angles. In these graphs, angles are represented as nodes and correlation scores as edges, the node sizes are proportional to the number of links, and the edge sizes are proportional to the correlation scores. Second, we carried out a principal component analysis (PCA)^53^ of the correlation matrices (specifically an eigen-decomposition after double-centering as described in previous work^33^). The positions of the dihedral angles were plotted in the space formed by the first three dimensions of the PCA.

### Collective variables

The observation of a set of *n* correlated dihedral angles *Χ*_*i*_ prompted us to define the collective variable *COV1*_*n*_ to monitor the transition associated to these *n* observables as:

6 $$ COV1_{n} (t) = \frac{{\sum\limits_{{{\text{X}}i = 1}}^{n} {\alpha_{{{\text{X}}i}} f_{{{\text{X}}i}} (t)} }}{{\sum\limits_{{{\text{X}}i = 1}}^{n} {\alpha_{{{\text{X}}i}} } }} $$

where α_*Χi*_ is the coordinate of the dihedral angle *Χ*_*i*_ on the first component and *f*_*Χi*_*(t)* is a delta function of time *t* which is equal to zero and to one when the rotameric state of *X*_*i*_ at time *t* corresponds, respectively, to the state observed before and after its rotational transition.

We also defined the collective variable *COV2* as the normalized distance between the Cα atoms of residues at positions 3.50 in TM3 and 6.30 in TM6. Normalization was based on the average values of the TM3-TM6 distance before and after the opening motion of TM6. *COV2* monitors the activation-like transition associated with the opening motion of TM6.

## Results

### A large conformational change is observed in CXCR4 simulations

**Figure 2:**
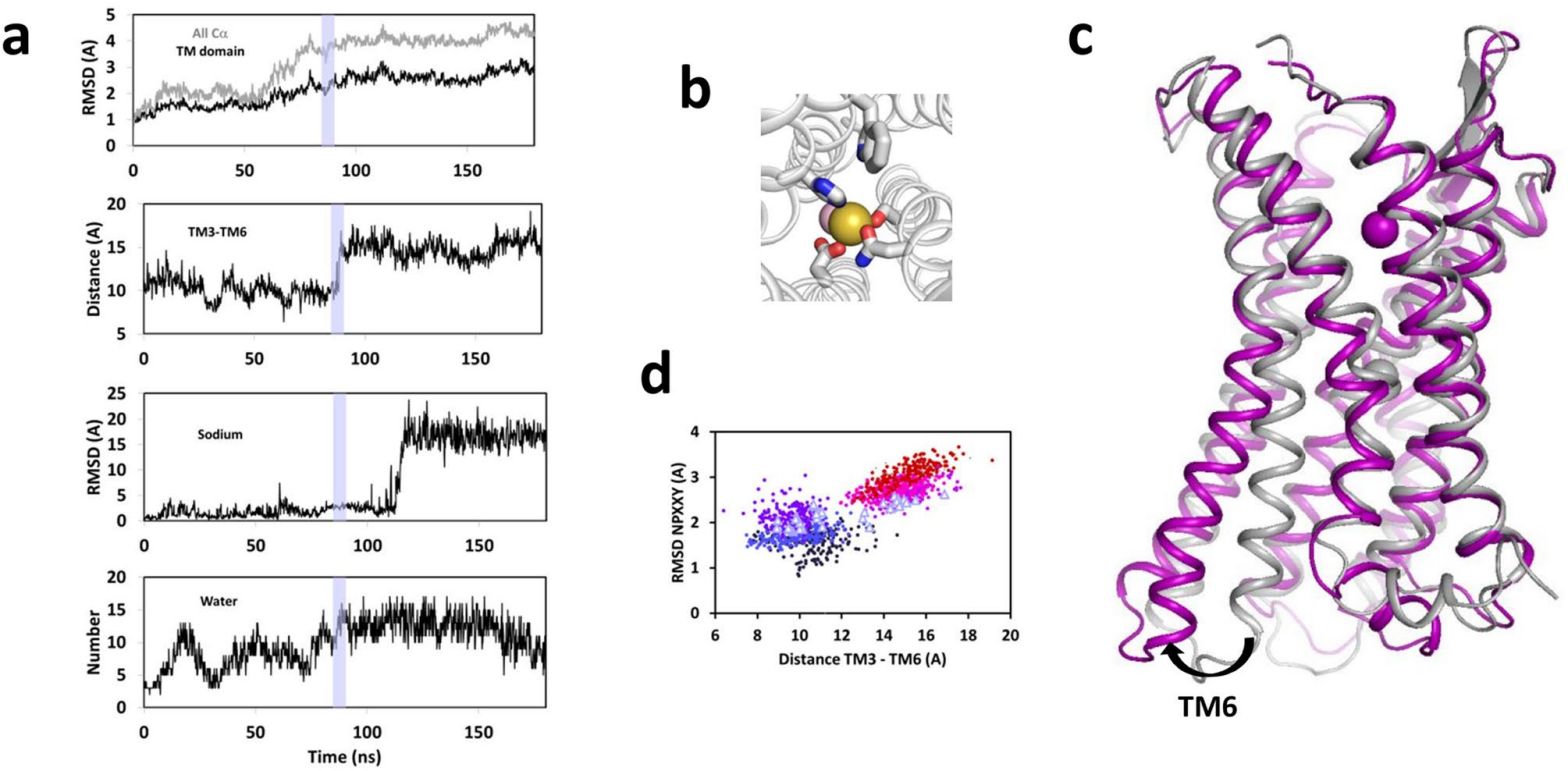
A large conformational change of CXCR4 occurs during a 180 ns aMD trajectory. (**a**) Time evolution of (from top to bottom): the RMSD of all the Cα atoms of the receptor (black line) and of the transmembrane domain only (grey lines); the distance between TM3 and TM6, measured as the distance between the Cα atoms of residues R134^3^^.^^50^ and K234^6^^.^^30^; the RMSD of the sodium ion; the number of water molecules within 6 Å of the Oδ1 or Oδ2 atoms of D84^2^^.^^50^; (**b**) Zooming on the canonical sodium binding site of CXCR4, as observed after 35 ns of simulation. The sodium (yellow sphere) is coordinated to the sidechains of D84^2^^.^^50^, N119^3^^.^^35^, S123^3^^.^^39^, H294^7^^.^^45^ and to a water molecule (pink sphere); (**c**) Superposition of the CXCR4 backbone at the beginning (grey ribbon) and at the end of the aMD simulation (purple). The sodium ion is shown as a sphere. The large outward motion of TM6 characterizes an activation-like transition; (**d**) 2D plot of the RMSD of the NPXXY motif in TM7 as a function of the distance between TM3 and TM6. The 180 ns trajectory is divided in six equal slots (color code from time 0 to 180 ns: dark blue, indigo, purple, violet, magenta and red dots). The transition frames from 85 to 90 ns are highlighted by triangles and correspond to the slate area in panel (**a**).

We have previously carried out molecular dynamic simulations of sodium-bound CXCR4 embedded within a POPC membrane. In this previous work, CXCR4 was modelled in an inactive state and was very stable during more than 400 ns of classical MD simulations^33^. In the present work, in order to expand the conformational space sampled by this receptor, we carried out aMD simulations that reduce energy barriers between states and facilitate conformational transitions^27^. During a 180 ns trajectory of aMD simulations, the RMSD of the receptor Cα atoms revealed two successive steps of conformational changes (Fig. 2a).

The first step corresponded to an equilibration of the structure to the acceleration conditions, with a very fast phase shorter than 1 ns, followed by a slower phase that reached a plateau after 20 ns. This step was accompanied by “breathing” of the receptor with water entrance and egress in the intracellular cavity (from 3 to 10 water molecules in the neighborhood of D2.50). At the beginning of the simulation and upon water egress (e.g., after 35 ns of simulation in Fig. 2b), the sodium ion was tightly bound to its canonical site^33^ that is formed by one water molecule and four protein atoms: the Oδ1 atom of D84^2^^.^^50^, the Oδ1 atom of N119^3^^.^^35^, the Oγ atom of S123^3^^.^^39^, and the Nε2 atom of H294^7^^.^^45^. The sidechains of D84^2^^.^^50^ and H294^7^^.^^45^ form the “bottom” of the sodium binding site, whereas N119^3^^.^^35^ closes it as a tap.

The second step was initiated after 60 ns and reached a plateau about 100–120 ns after the beginning of the acceleration. The characteristic outward motion of TM6, typical of an active-like transition, occurred during this second phase after 90 ns of simulation (Fig. 2c). The motion of TM6, quantified by the increased distance between TM3 and TM6 from 10 to 18 Å, was accompanied by a reorganization of the NPXXY motif (Fig. 2d). After 20 ns, this conformational change was followed by the egress of the sodium ion towards the extracellular cavity of the receptor (Fig. 2a,c). It is worth noting that water entrance and backbone RMSD preceded the outward motion of TM6.

### ***CIRCULAR*** and ***OMES*** scores highlight a few correlated sidechain pairs

**Figure 3:**
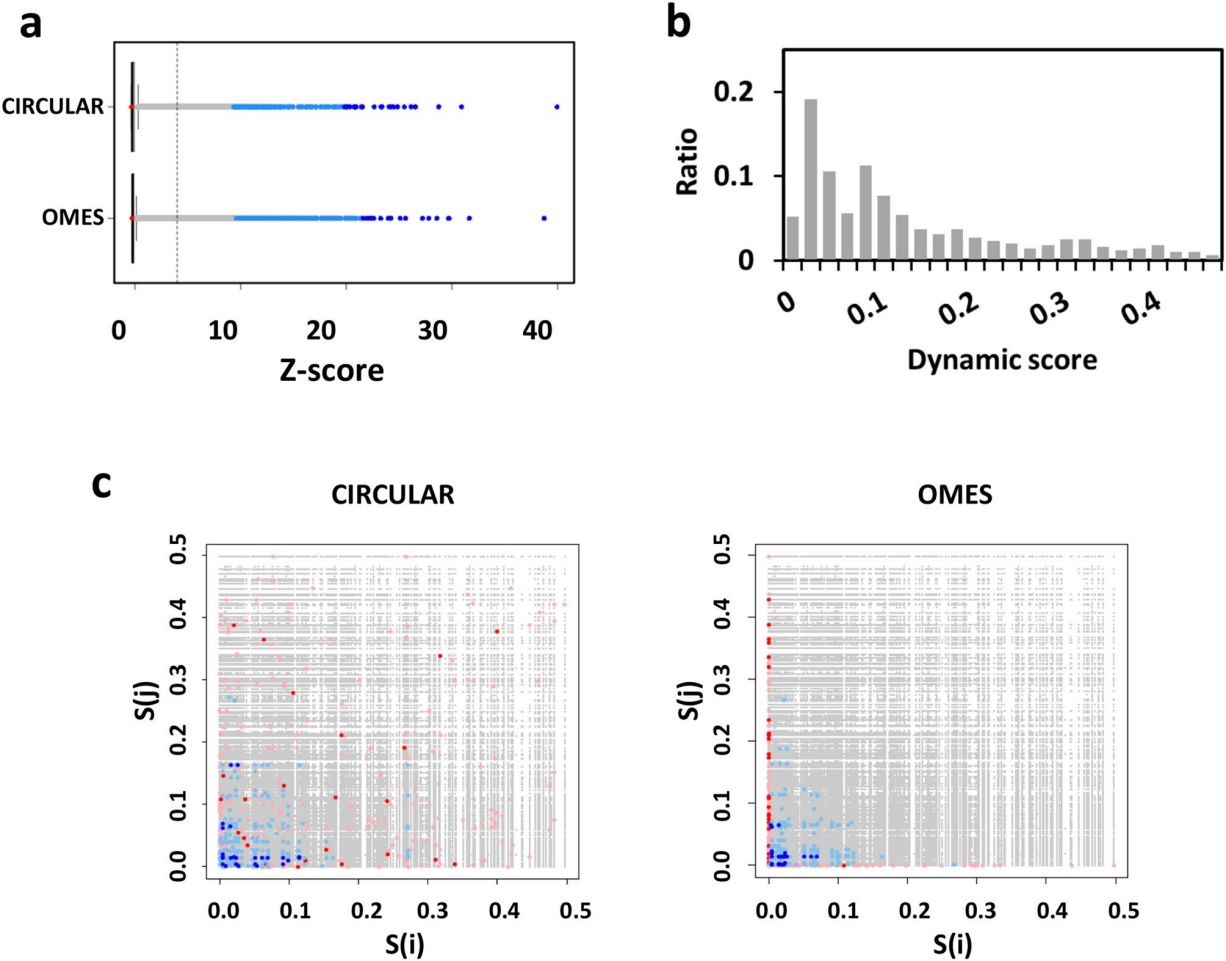
Correlation scores highlight a few dihedral angle pairs. (**a**) Boxplots of the *Z*-scores obtained with the *CIRCULAR* and *OMES* methods; (**b**) Histogram of the dynamic scores *S* of the sidechain dihedral angles. The left bar indicates the ratio of dihedral angles with no rotamerization (*S* = 0), then the following bars correspond to increasing *S* by 0.02 range; (**c**) Dependence of the *Z*-scores on the dynamic scores *S* of the dihedral angles (*CIRCULAR*: left, *OMES*: right). In (**a**) and (**c**), the top 25 pairs are dark blue, the next 250 pairs are light blue, the 25 and 250 bottom pairs are, respectively, red and pink.

Correlation scores between sidechain dihedral angles were computed with both the *CIRCULAR* function, based on dihedral values, and the *OMES* function, based on rotamer distributions. Boxplots of the *Z*-scores revealed positively skewed distributions, in agreement with the *CIRCULAR* and *OMES* formula, a bulk of close-to-zero scores and about 1% of the pairs with *Z*-scores larger than 4. Both methods highlighted a few pairs with very large *Z*-scores, reaching up to a value of 40 (Fig. 3a).

To assess the significance of these scores, we analyzed the relationship between the correlation scores and the motility of the sidechains. The motility of a dihedral angle χ was estimated by the “dynamic score” function, *S*(χ), which measures the number of rotameric interconversions during the trajectory, normalized by the number of frames. The theoretical maximum value of the dynamic score is 0.66, when a dihedral angle randomly rotamerizes between the three possible rotameric states^54^. In the trajectory under investigation, 5% of the dihedral angles did not rotamerize whereas the probability of rotamerization varied from less than 0.02 to more than 0.10 for 20% and 50% of the dihedral angles, respectively (Fig. 3b). We plotted the correlation scores as a function of the dynamic scores of each angle in the pair (Fig. 3c). Most angles involved in the top 25 pairs had low dynamic scores, with average values of 0.027 and 0.044 for *OMES* and *CIRCULAR*, respectively. Nevertheless, a few angles involved in top 25 pairs had dynamic scores of about 0.100 or higher with *CIRCULAR*. The dynamic scores of angles in bottom ranking pairs were variable with *CIRCULAR* and equal to 0 with *OMES*. For both methods, the top pair with the highest score involved the chi1 angles of N119^3^^.^^35^ and H203^5^^.^^42^.

### A set of correlated sidechains rotamerize just before or during the transition

**Figure 4:**
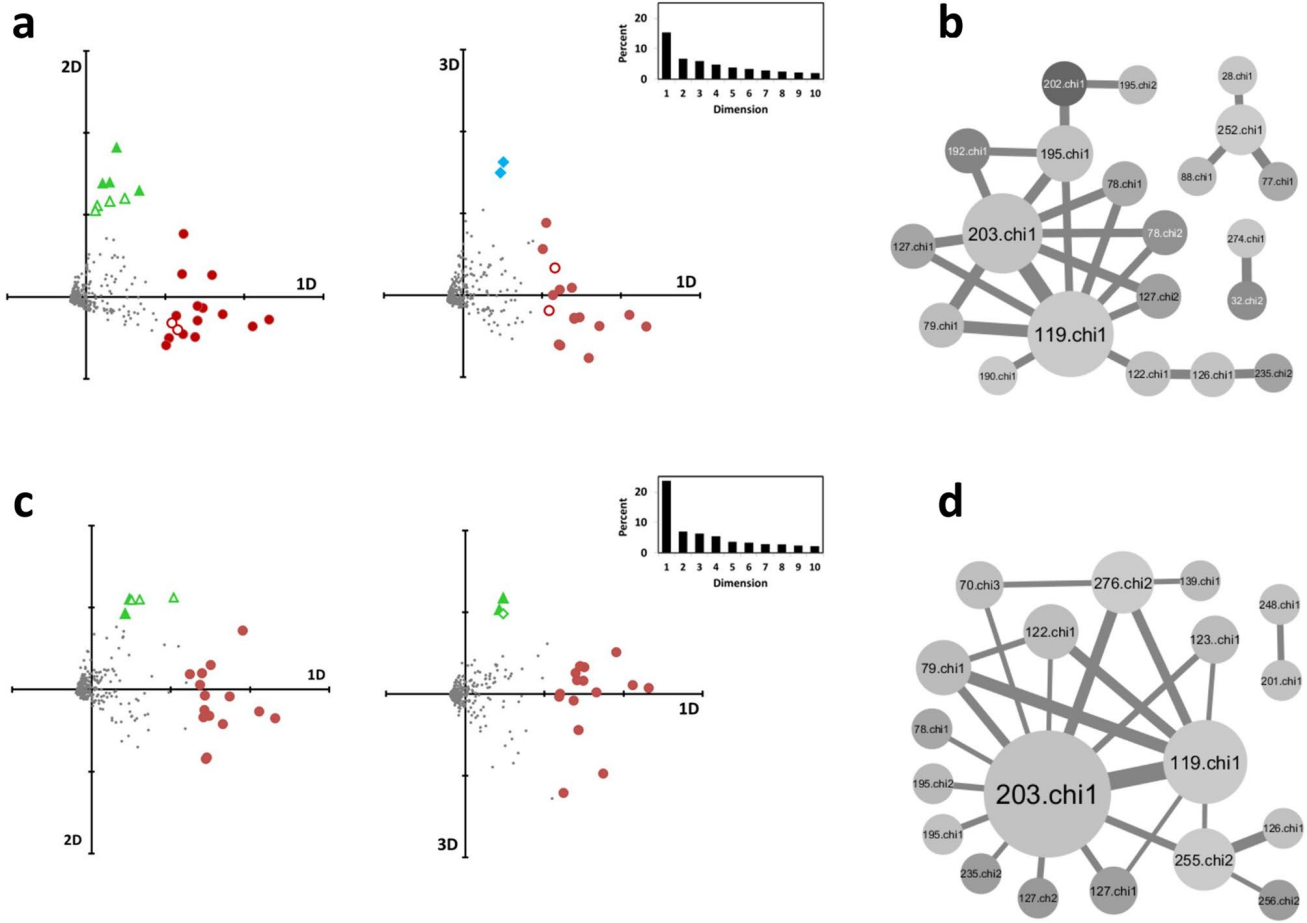
Eigen decomposition separates independent events. (**a**,**b**) Projection of the dihedral angles onto the principal components of the *CIRCULAR* (**a**) and *OMES* (**b**) correlation matrices; (**c**,**d**) Network representation of the top 25 scores obtained with *CIRCULAR* (**c**) and *OMES* (**d**). The node size is proportional to the number of links. The node color depends on the dynamic score *S* from light grey for *S* = 0 to dark grey for *S* = 0.16. The edge size is proportional to the correlation score. The top PCA positions present and absent in the networks of the top 25 pairs are indicated by, respectively, closed and open symbols (red circles for the largest network, green triangles for the second largest one and cyan diamonds for the smallest one).

The eigen decomposition of the double-centered correlation matrices was carried out according to a previously developed procedure^33^ and the dihedral angles were projected onto the principal components (Fig. 4a,c). This analysis highlighted a few angles clearly separated along the first three principal components. In both cases, the dihedral angles H203^5^^.^^42^.chi1 and N119^3^^.^^35^.chi1 had the highest coordinates on the first component. This analysis was consistent with the network representation of the top 25 pairs. Angles spread along the first component were involved in the main network of correlated angles, while angles spread along the second and third components were involved in secondary networks.

The network representation of the top pairs (Fig. 4b,d) revealed differences between the methods. *CIRCULAR* displayed a main network, with a strong link between N119^3^^.^^35^ and H203^5^^.^^42^ (with 6 common contacts out of a total of 9 and 7 contacts, respectively), but also a secondary network around the chi1 angle of W252^6^^.^^48^. *OMES* favored an overwhelming, hub-like network with central H203^5^^.^^42^ (13 contacts), strongly linked to N119^3^^.^^35^ (6 contacts, also linked to H203^5^^.^^42^). These representations agreed with the proportion of the variance explained by the first component in *OMES* and *CIRCULAR* (25 and 15%, respectively).

**Figure 5:**
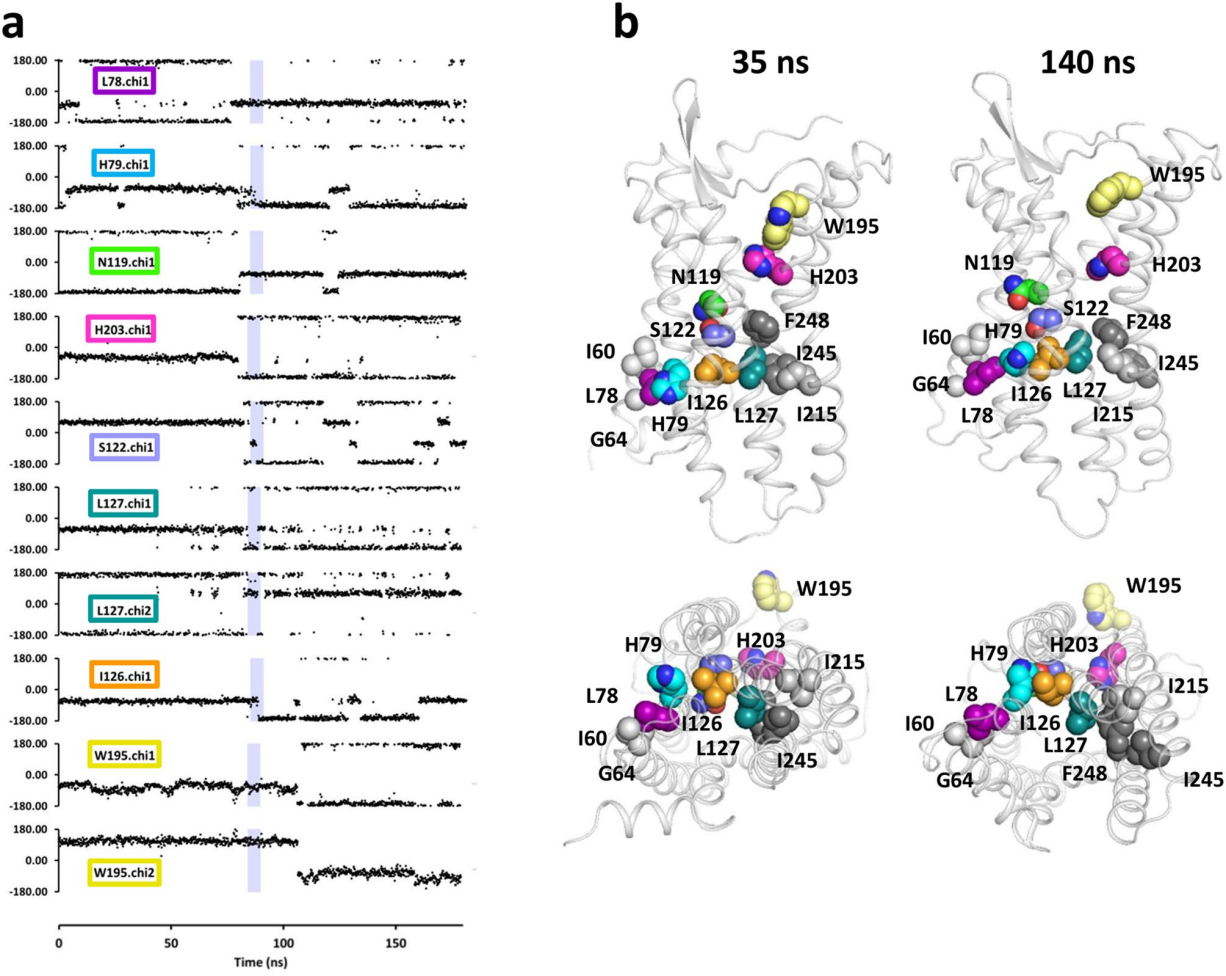
Consensus angles rotamerize just before or during the transition. (**a**) Time evolution of the top correlated angles obtained with both *CIRCULAR* and *OMES*. The angles are sorted by their rotamerization time. The slate areas indicate the TM6 outward motion (85–90 ns). (**b**) Positions of the residues with top correlated angles in the 3D structure of the receptor before the transition (35 ns) and after the transition (140 ns). Top and bottom panels are viewed from, respectively, the lateral and intracellular side. The residues are shown as spheres with the following color code: L78^2^^.^^44^, purple; H79^2^^.^^45^, cyan, N119^3^^.^^35^, green; S122^3^^.^^38^, slate; I12^63^^.^^42^, orange; L127^3^^.^^43^, deep-teal; W195^5^^.^^34^, yellow; H203^5^^.^^42^, magenta. The residues in TM1, TM5 and TM6 in contact with L78^2^^.^^44^ and L127^3^^.^^43^ are also shown as spheres (I60^1^^.^^54^ and G64^1^^.^^58^, light grey; I215^5^^.^^54^, middle grey; I245^6^^.^^41^ and F248^6^^.^^44^, dark grey spheres).

The time evolution of all the highlighted angles is shown in Fig. 5a and in Supplementary Fig. S1. Briefly speaking, most angles observed on the first component with *OMES* or *CIRCULAR* underwent a rotamerization event around 80 ns, as exemplified by the ten consensus angles that are found in the top 25 pairs of both methods (Fig. 5). The *CIRCULAR* second component highlighted angles that rotamerized with W252^6^^.^^48^ at the end of the simulation and the third component highlighted a few angles with a double rotamerization. The second/third components with *OMES* also highlighted angles with a double rotamerization. These results indicate that independent rotational events are visualized on different principal components.

Focusing on the consensus angles from the first component, we can note that eight out of the ten angles rotamerized within a short time window from 76 to 88 ns, immediately before or during the opening motion of TM6. The only exceptions to this pattern were the dihedral angles of W195^5^^.^^34^, whose rotation was delayed by about 25 ns. As this residue is directed towards the outside without contact with the other residues, its high scores appear as a fortuitous consequence of its low dynamic score (Fig. 5b) and it has been excluded from subsequent analysis.

Within the 12 ns window, the sequential order of the events was as follows: (1) The rotamerization of L78^2^^.^^44^.chi1 was followed, after 3 ns, by the instability of H79^2^^.^^45^.chi1 that underwent frequent interconversions between the *g*– and the *trans* rotamers during 6 ns; (2) One ns after the beginning of the H79^2^^.^^45^.chi1 instability, N119^3^^.^^35^.chi1 rotamerized; (3) This rotamerization was followed by the rotamerization of H203^5^^.^^42^.chi1 after 0.3 ns, S122^3^^.^^38^.chi1 after 2 ns (with interconversions between the *trans* and *g-* rotamers up to the end of the TM6 opening), and L127^3^^.^^43^.chi1 and .chi2 after 3 ns; (4) Finally, the rotamerization of I126^3^^.^^42^.chi1 occurred after 12 ns, at the end of the TM6 outward motion.

### Highlighted sidechains are involved in the mechanism of receptor activation

The position of the seven residues undergoing these correlated rotamerizations can be visualized on the structure of the receptor (Fig. 5b). The six residues located on TM2 (L78^2^^.^^44^, H79^2^^.^^45^) and TM3 (N119^3^^.^^35^, S122^3^^.^^38^, I126^3^^.^^42^ and L127^3^^.^^43^) formed a cluster of neighbor residues. The seventh residue, H203^5^^.^^42^, pointed towards TM3 and TM6, before and after the transition, respectively. L78^2^^.^^44^ was involved in packing with TM1 (I60^1^^.^^54^ sidechain and G64^1^^.^^58^ Cα atom), while L127^3^^.^^43^ was involved in packing with TM5 and TM6. In particular, L127^3^^.^^43^ was tightly involved in van der Waals interactions with I245^6^^.^^41^ and F278^6^^.^^44^, before the transition, and with I215^5^^.^^54^ and F278^6^^.^^44^, after the transition. Reorganization of helix packing involving the residue at position 6.44 has been observed upon receptor activation^21^.

**Figure 6:**
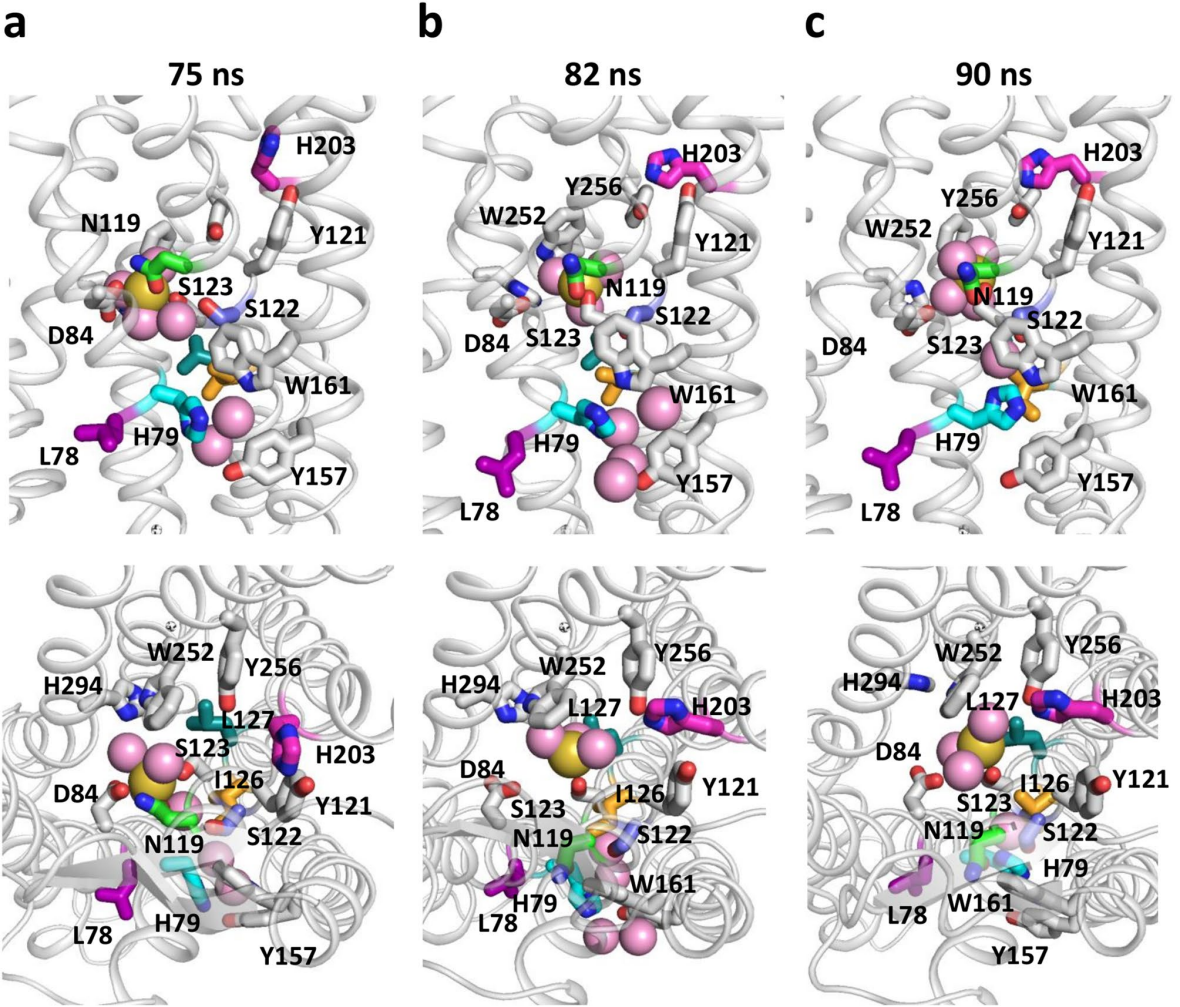
Rotamerization allows reorganization of the interactions. Three snapshots at 75 ns (**a**), 82 ns (**b**) and 90 ns (**c**) have been selected to show the reorganization of the interactions involving the consensus residues during the activation-like transition. Top and bottom panels are viewed from the lateral and extracellular sides, respectively. The top correlated residues are shown as sticks with the following color code: L78^2^^.^^44^, purple; H79^2^^.^^45^, cyan, N119^3^^.^^35^, green; S122^3^^.^^38^, slate; I126^3^^.^^42^, orange; L127^3^^.^^43^, deep-teal; H203^5^^.^^42^, magenta. Neighbor sidechains are shown as white sticks. The sodium ion is shown as a yellow sphere, water molecules in coordination with the sodium ion or in the vicinity of H79^2^^.^^45^ and W161^4^^.^^50^ are shown as pink spheres. For clarity purpose, a scale of 0.7 is used for the spheres.

To decipher the functional role of these residues, we analyzed their interactions throughout the activation process. Three representative snapshots, just before the beginning of the rotamerization cascade (*t* = 75 ns), just before the beginning of the TM6 motion (*t* = 82 ns) and at the end of the TM6 motion (*t* = 90 ns) are shown in Fig. 6. During the first 75 ns, N119^3^^.^^35^ stably interacted with the sodium ion. However, in the aMD simulation, receptor breathing led to water permeation within the receptor core (Fig. 2a). This altered the sodium binding mode by substitution of H294^7^^.^^45^ coordination^33^ by water. Just before the rotamerization events (Fig. 6a), the sodium ion interacted with D84^2^^.^^50^, N119^3^^.^^35^ and S123^3^^.^^39^, and three water molecules. In addition, H203^5^^.^^42^ was H-bonded to Y121^3^^.^^37^.

The cascade of rotamerizations initiated with L78^2^^.^^44^. Rotation of L78^2^^.^^44^ and subsequent instability of H79^2^^.^^45^ led to water permeation in the cleft between TM2 and TM4, which suggests a destabilization of the TM2-TM3-TM4 fold. In addition, just before the rotamerization of N119^3^^.^^35^, a water molecule moved from the first sodium shell to the outward face of TM3, in the vicinity of S122^3^^.^^38^ (Supplementary Fig. S2). The presence of this water molecule may have facilitated the subsequent outward rotamerization of N119^3^^.^^35^ toward S122^3^^.^^38^. The N119^3^^.^^35^ rotamerization was followed by water escape and then by the rotamerization of S122^3^^.^^38^ and L127^3^^.^^43^. The N119^3^^.^^35^ rotamerization strongly disrupted the canonical sodium binding site. The sodium moved by about 4 Å (Fig. 2a) towards a secondary binding site where it interacted with S123^3^^.^^39^ and with the highly conserved W252^6^^.^^48^ through a water molecule or cation-π interaction^55^. Concomitantly, H203^5^^.^^42^ also rotamerized. This residue has also been identified as important for signal transmission in a comprehensive library of CXCR4 mutants^56^. Its new orientation towards TM6 was stabilized by an H-bond with Y256^6^^.^^52^. The reorganization of the H-bonding pattern of H203^5^^.^^42^ from TM3 to TM6 might help the reorganization of the interactions between the helices. This cascade of events was completed at *t* = 82 ns (Fig. 6b) and preceded the wobbling motion of TM6 which occurred when the sodium ion was located in the secondary site.

At the end of the transition (Fig. 6c), the stabilization of H79^2^^.^^45^ in the *trans* conformer and the rotamerization of I126^3^^.^^42^ led to a stable cluster involving H79^2^^.^^45^, W161^4^^.^^50^, I126^3^^.^^42^, S122^3^^.^^38^, and N119^3^^.^^35^. The sodium ion remained in the vicinity of S123^3^^.^^39^ for about 20 ns and subsequently escaped towards the extracellular cavity of the receptor at time *t* = 112 ns (Fig. 2). Egress of sodium is a characteristic feature of receptor activation and might lock the receptor active state^57–59^.

## Discussion

Large scale conformational changes are complex combinations of local and global changes. Local reorganization of intramolecular interactions is a key step in the molecular processes yielding global changes. The increase in computational power along with the development of methods aimed at accelerating MD simulations have made it possible to observe conformational transitions during MD simulations. However, the interpretation of the MD data in terms of molecular mechanisms is not straightforward, because of the very high number of variables, and because it is hard to decipher the precise mechanistic details on how residues functionally cooperate. This problem prompted the development of a variety of methods aimed at dimensionality reduction^60,61^. Among these methods, PCA analysis^53^ of the Cartesian coordinate covariance matrix has been widely used to determine collective variables. Collective variables could also be obtained from internal coordinates such as backbone dihedral angles or inter-residue distances^62,63^. The choice of the “best” coordinates for PCA analysis depends on the system investigated and may require “manual” inspection of the raw data^64^.

Sidechain dihedral angles did not receive the same attention as backbone angles because they may undergo numerous rotamerizations during a trajectory. Nevertheless, compared to inter-residues or contact distances, these angles present an advantageous feature because their values depend not only upon direct van der Waals contacts or H-bonds with other protein or ligand atoms but also upon indirect H-bond interactions through water molecules or ions. Thus, the analysis of dihedral angles may usefully complement other approaches to identify allosteric sites for drug binding or mechanisms of conformational transition.

In this paper, we developed a method aimed at determining (quasi-)simultaneous isomerization events of sidechains in a MD trajectory to gain information on protein conformational transitions. When applied to a transition from an inactive to an active-like state of CXCR4, this new method reveals several points of general use:

1. The largest correlation scores observed with *CIRCULAR* correspond to rotamerization events and overlap those observed with *OMES*. The consistency between *CIRCULAR* and *OMES* results indicates that the periodic nature of the dihedral angles is correctly taken into account in the sinus space analyzed in Eq. (3)—an effect that may be due to the presence of rotamers with well separated average sinus values.
2. For each method, the network and the PCA approaches give similar results. PCA presents the advantage of avoiding a user–defined parameter. Nevertheless, the choice of 25 top ranking pairs, representing about 5% of the 480 dihedral angles analyzed, allows a fair consistency between network and PCA representations of the data, with clusters corresponding to positions spread along the three principal components.
3. Most angles in top ranking pairs have rare rotamerizations during the trajectory. However, some angles may present instability during a part of the trajectory, transient changes to another rotameric state or secondary transitions (see examples in Fig. 5a). Limiting the analysis to residues with a number of rotamerizations below a cut-off value might prevent highlighting interesting “true” correlations.
4. The main differences between *CIRCULAR* and *OMES* are similar to those observed between the *McBASC* and the *OMES* methods for sequence analysis^50^. *OMES* favors a hub-like, overwhelming network, whereas *CIRCULAR* is more successful in highlighting independent co-variations (see Supplementary Fig. S1). As a matter of fact, the first principal component represents, respectively, for *OMES* and *CIRCULAR*, about 25% and 15% of the data covariance. So, *CIRCULAR* might be the first choice for exploring a whole trajectory, whereas *OMES* might lead to a wider coverage of correlated rotamerizations in link with a single transition in (part of) a trajectory. Consensus results should shed light on important sidechain motions in complex multistep transitions.
5. More subtle differences are observed, mainly for the second or the third principal components. *CIRCULAR* displays more variability in highlighted angles. For example, the 202.chi1 angle interconverted between the (*trans, g* +) and (*trans*, *g*–) rotamers before and after the transition, respectively. By contrast, the 190.chi1 angle remained in the same rotameric state, but its average values changed from – 80 ± 11° to – 62 ± 10° upon the transition (Fig. S1).

Concerning the application of *CIRCULAR* and *OMES* to our case study (i.e., an activation-like transition of CXCR4), the analyses highlighted eight dihedral angles that underwent a rotamerization within a 12 ns window just before or during the conformational transition—a pattern that strongly suggests that these rotamerizations might be important for the receptor conformational change. These rotamerizations were not *stricto senso* “simultaneous,” but occurred in a sequential order that could be rationalized in terms of reorganization of the interactions. Thus, these rotamerizations preceded (e.g. N119^3^^.^^35^) or accompanied (e.g. L126^3^^.^^43^) the transition from an inactive to an active-like conformation.

**Figure 7:**
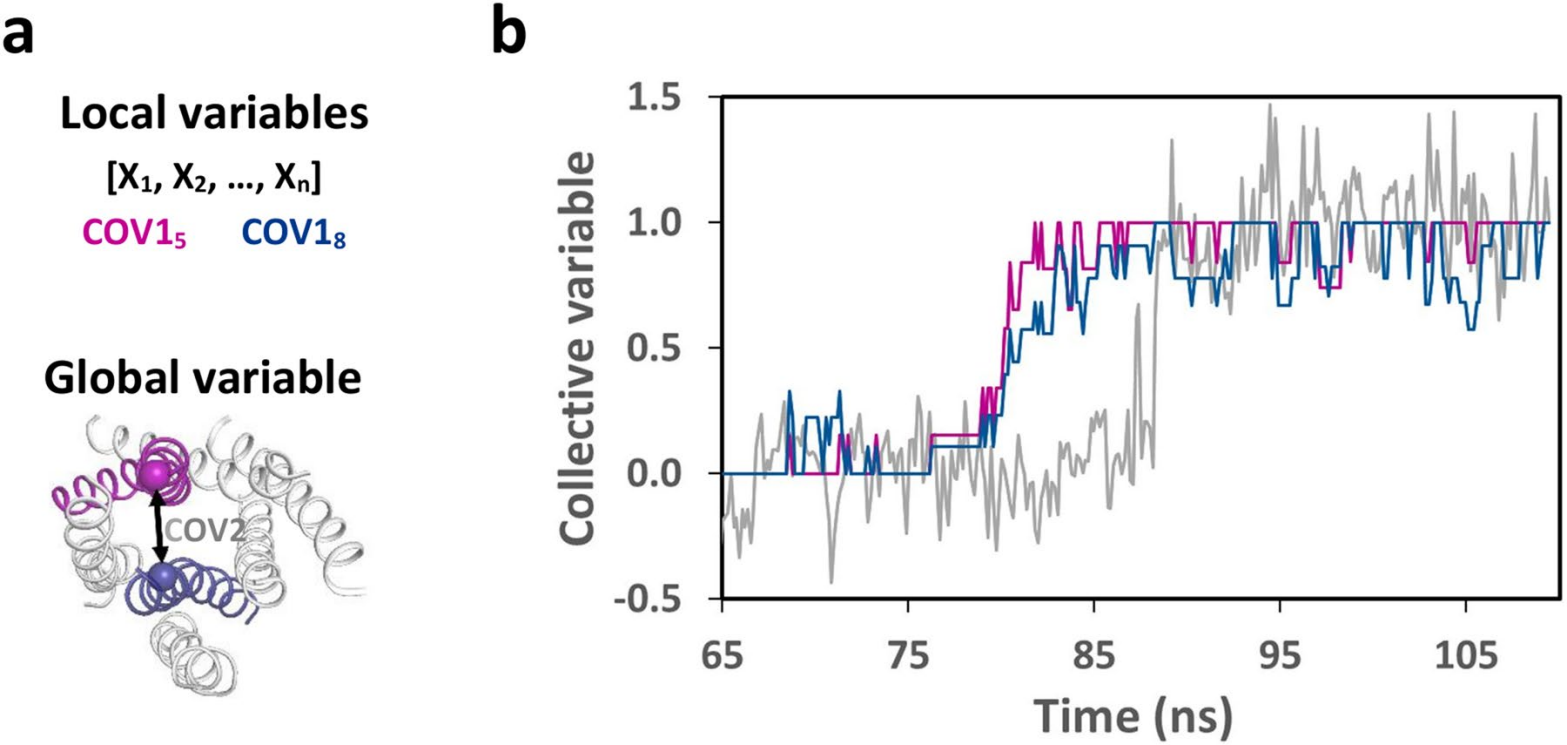
Collective variables highlight the delay between local and global changes. In (**a**), schematic representation of the collective variables that describe local and global changes. The *COV1* variables describe the rotational motions of either the consensus eight angles (COV1_8_) or of the five angles that first rotamerize (*COV1*_5_). The *COV2* variable measures the distance between the Cα atoms of R134^3^^.^^50^ in TM3 (in blue) and K234^6^^.^^30^ in TM6 (in purple) in a ribbon representation of the seven TM bundle. In (**b**), time evolution of the collective variables COV1_5_ (purple), COV1_8_ (blue) and COV2 (grey).

To further analyze these events, we defined the collective variables *COV1*_*8*_ and *COV1*_*5*_ in Eq. (6) to monitor the rotational transitions associated with, respectively, the eight dihedral angles under scrutiny and the five dihedral angles that first rotamerized. In Fig. 7, they were compared to *COV2*, the collective variable describing the activation-like transition of CXCR4. The 8 ns delay between the first five rotamerizations and the activation-like transition strongly suggests an allosteric mechanism in which the local changes monitored by *COV1*_*5*_ facilitate the subsequent global change measured by *COV2*.

Among the interconverting residues, the rotamerization of N119^3^^.^^35^ appears to be a key step for receptor activation—an effect consistent with functional and structural data. First, the mutation of N119^3^^.^^35^ to Ser or Ala confers constitutive activity to CXCR4^65^, supporting a role of negative modulator for N119^3^^.^^35^. Second, in the inactive structures of CXCR4^34^ and related receptors, such as the μ opioid receptor^35^, the angiotensin II (AngII) receptor AT1^66^ and the protease-activated receptor 1^67^, N3.35 is in the *trans* conformation. In this inward orientation, it can participate in the coordination of the sodium ion and thus in the stabilization of the inactive state^68,69^. By contrast, the *g*– rotamer of N3.35, directed outward, has been observed in the active conformation of three CXCR4 related receptors, the μ opioid receptor^70^, and the AngII receptors AT1^71^ and AT2^72,73^. These data suggest that the orientation of N3.35 may be an important feature of receptor activation. Finally, by extensive MD simulations of diverse active forms of AT1, Lefkowitz and co-workers^74^ have shown evidence that the orientation of N3.35 plays a major allosteric role in receptor function. The outward orientation of N3.35 favors the canonical active conformation of AT1 prone to G protein signaling whereas the inward orientation favors an alternative active conformation prone to β-arrestin signaling. Taken together, these data strongly suggest that the outward motion of N119^3^^.^^35^ that we observe during the CXCR4 simulation plays an important allosteric role in the activation of this receptor.

It is important to note that water permeation within the transmembrane fold preceded these events and played an important role in the subsequent steps. First, water entrance (Fig. 1a) modified the coordination of the sodium ion with substitution of H294^7^^.^^45^ by water molecules, which is expected to weaken the sodium binding site. Second, water could directly favor sidechain motion. As an example, Supplementary Fig. S2 shows that the outward rotamerization of N119^3^^.^^35^ occurred when a water molecule was located near S122^3^^.^^38^ on the outward face of TM3 and that the escape of the water molecule was followed by the rotamerization of S122^3^^.^^38^. Water is an important element of GPCR activation^22^ and, indeed, GPCR activation during aMD simulations can be observed with the dual boost procedure which “accelerates” water molecules but not with the single boost procedure which acts only on dihedral angles^27^.

It is also important to note that the rotamerization of N119^3^^.^^35^ was immediately followed by the rotamerization of H203^5^^.^^42^ (a residue involved in signal transmission^56^) and then by a reorganization of H-bonding pattern of this latter residue from TM3 to TM6—a reorganization modifying the interactions stabilizing the receptor structure. Reorganization of packing interaction upon the transition was also observed for the packing of L78^2^^.^^44^ with TM1 and of L127^3^^.^^43^ with TM5 and TM6 (Fig. 5). The role of L127^3^^.^^43^ in the transition pathway is consistent with the constitutive activation of several receptors upon mutation of this position^75^.

The N119^3^^.^^35^ rotational motion allowed the sodium ion (1) to escape its canonical binding site, strongly altering the stability of the inactive structure and (2) to move to a secondary site in the vicinity of W252^6^^.^^48^. This latter residue is an important molecular switch for receptor activation^19,76^. The observation of the conformational transition when the sodium ion is in the vicinity of W252^6^^.^^48^ suggests that the sodium ion might have a dual role, acting, respectively, as negative and positive allosteric modulator, in the canonical and in secondary sites. The dual effect of sodium on receptor activation has been observed for opioid receptors^59,77,78^ and might be a general feature of class A GPCRs^69^. Finally, the role of the N119^3^^.^^35^ rotamerization in the activation suggests that the cleft between TM2 and TM4 might be an allosteric site, with ligands favoring the rotamerization of N119^3^^.^^35^ toward the membrane acting as positive allosteric modulators. This effect is reminiscent of the agonist role of pepducins that can activate CXCR4 receptor through an intracellular mechanism^79,80^.

In conclusion, applied to a spontaneous transition of CXCR4, observed during a 180 ns aMD simulation, our method (1) highlights “collaborative” sidechain motions that immediately preceded the conformational transition and (2) suggests an allosteric mechanism involving N3.35 that makes sense for a receptor with this sequence motif. However, these results do not imply that there is a unique transition pathway for CXCR4 activation. Indeed, enhanced MD simulations of GPCR activation using adaptive sampling or biased MD simulations suggest the existence of multiple transition pathways^12,26^. This pattern is consistent with the dynamic character of protein structures and the flexibility of the interactions within a protein that may be best described using network formalism^81,82^. In any case, the present analysis provides the proof-of-concept that the correlation method developed here is a valuable exploratory technique that helps decipher complex reactional pathways.

## Supplementary information

Supplementary Information.

## Data Availability

All the data used for this article have been deposited at the Mendeley Data Repository (https://doi.org/10.17632/5v7bmfctsz.1). The description of the deposited data is given in Supplementary File S1. The scripts developed for this study have been integrated into the R package Bios2cor (version 2.1) that is available at the Comprehensive R Archive Network (https://cran.r-project.org/web/packages/Bios2cor/index.html).
