# Supplementary material for: Deciphering collaborative sidechain motions in proteins during molecular dynamics simulations
Source: Sci Rep. 2020 Sep 28;10:15901. doi: 10.1038/s41598-020-72766-1 (PMC7522237; doi:10.1038/s41598-020-72766-1)

Deciphering collaborative sidechain motions in proteins during molecular dynamics simulations

Bruck Taddese, Antoine Garnier, Hervé Abdi, Daniel Henrion, and Marie Chabbert

File S1: Dataset description

All the data used for this article have been deposited at the Mendeley Data repository (doi:10.17632/5v7bmfctsz.1).

Title: Example of molecular dynamics simulations for analysis of sidechain correlations with the R package `bios2cor`.

Description

This dataset provides all the data necessary to analyze correlated sidechain motions in an accelerated molecular dynamics trajectory of CXCR4 with the `bios2cor` package. It includes:

(1) the raw trajectory with input and parameters files. The trajectory corresponds to a 180 ns accelerated molecular dynamics simulation of CXCR4 with bound sodium. Data includes: the cxcr4_assembly.pdb, cxcr4_assembly.xplor_ext.psf and checkftt.str files obtained with charmm-gui, the initial .coor, .vel and .xsc files for the aMD simulations, the namd configuration file and the resulting 180 ns trajectory (1200 frames with all atoms in the simulation).

(2) 25 snapshots from the 180 ns CXCR4 aMD trajectory. They are combined into a single pdb file. The snapshots are regularly spaced from frame 0 to frame 1200. The pdb file includes protein atoms, the sodium ion and water molecules within 3 Å from protein. The snapshots shown in Fig. 6 correspond to frames 500, 550, and 600 of the trajectory.

(3) all the data for the Bios2cor analysis: the version 2.1 of the package, the documentation, the input, output and script files for test. The input files correspond to the dcd trajectory and pdb file of frame 0 for protein and sodium atoms only (1200 frames). The 28 output files are created with the correlation_analysis.R script.

(4) an excel file giving the equivalence between CXCR4 numberings: the “true” sequence numbering, the Ballesteros’numbering and the numbering in pdb and dcd files (the CXCR4 model started at residue 1 corresponding to residue 28 in sequence numbering).

Fig S1: Time evolution of the correlated dihedral angles specific to CIRCULAR and OMES.

All the displayed angles are indicated by color symbols in Fig. 4a (circles, triangles and diamonds for angles separated on, respectively, the first, second and third components of the PCA analysis). Angles with black labels are also present in the top 25 pairs (closed symbols in Fig. 4a). Angles with grey labels are not present in the top 25 pairs (open symbols in Fig. 4a). The only exception to this pattern is the angle 256.chi2, found in the top 25 pairs obtained with OMES and not observed by PCA.

CIRCULAR specific angles on PC1

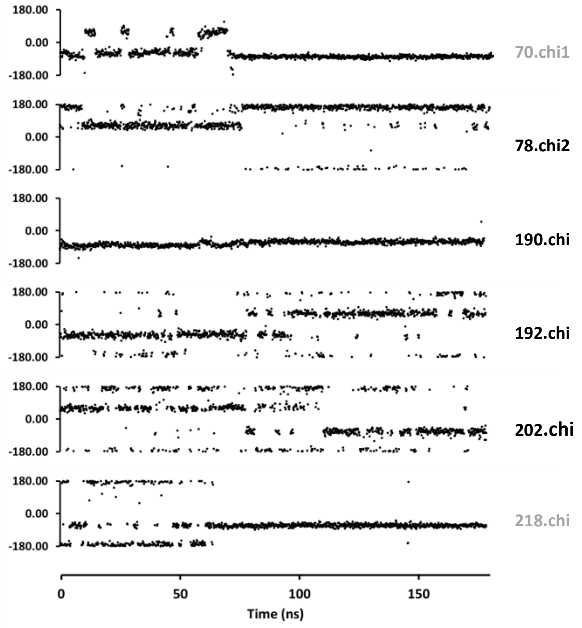

CIRCULAR specific angles on PC2

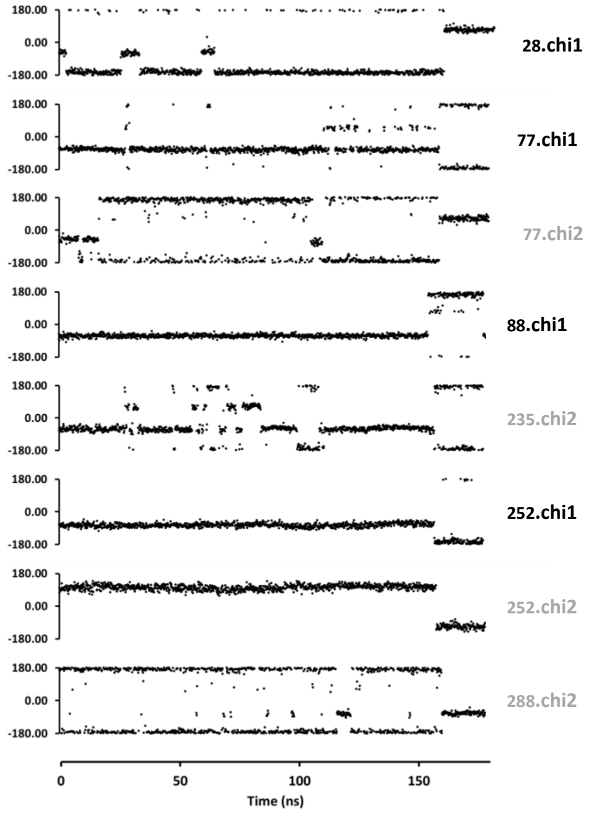

CIRCULAR specific angles on PC3

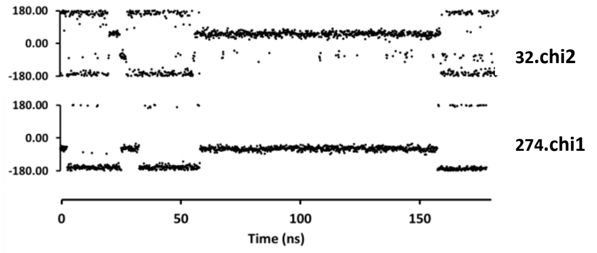

OMES specific angles on PC1

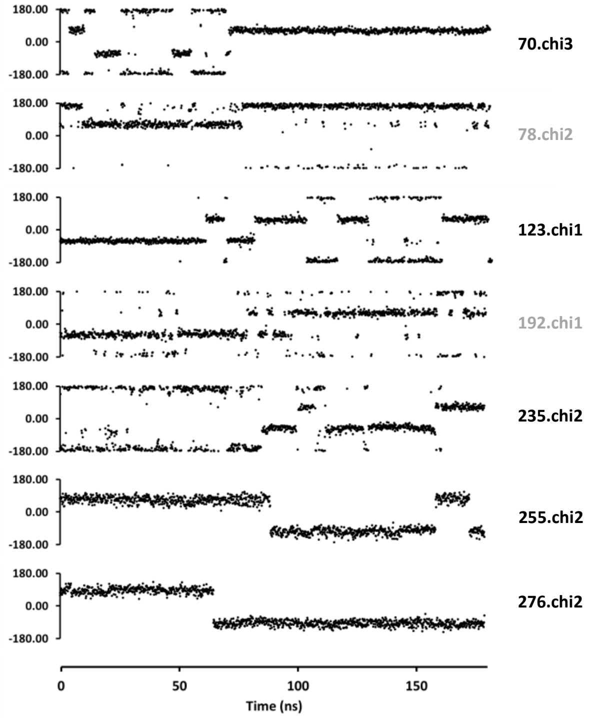

OMES specific angles on PC2 and PC3

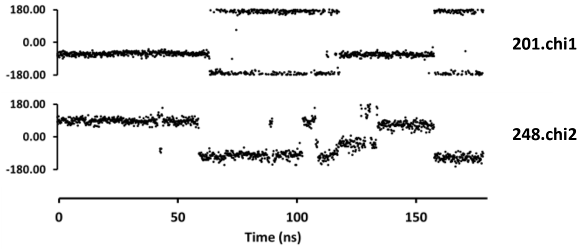

OMES specific angles on PC2 only

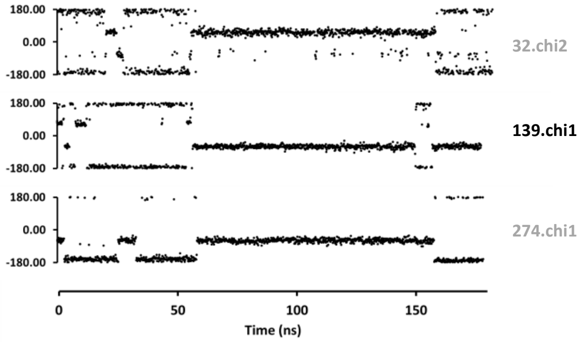

OMES specific in main network

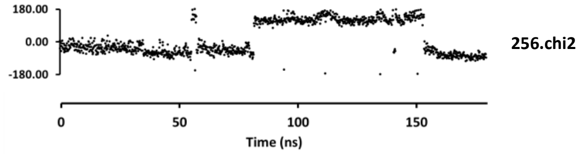

OMES specific angles on PC3 only

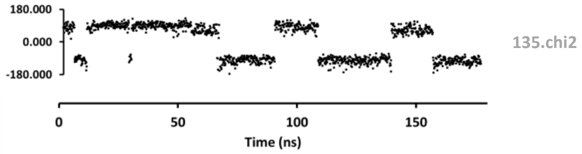

Fig S2: Role of water in sidechain rotational motions.

Six snapshots ranging from 73 to 82 ns follow the trajectory of a water molecule (shown as a red sphere) involved in the rotamerization of N3.35. In (a), at 73 ns, the water molecule enters into the first coordination shell of the sodium ion; In (b), 5 ns later, the water is still in the coordination shell of the sodium, at a different position; In (c), at time 79 ns, the water molecule moves from the sodium shell to the vicinity and S3.38 (slate), on the outward face of TM3; In (d), at time 80 ns, N3.35 (green) has rotamerized outward, towards S3.38 and the water molecule. This is followed in (e) by the water escape through the membrane facing cleft between TM2 and TM4 and then in (f), at time 82 ns, by the rotamerization of S3.38 which may be related to repulsive interaction between the OD1 atom of N3.35 and the OG atom of S3.38. Other water molecules filling the receptor interior are shown as pink spheres. The sodium ion is shown as a yellow sphere.

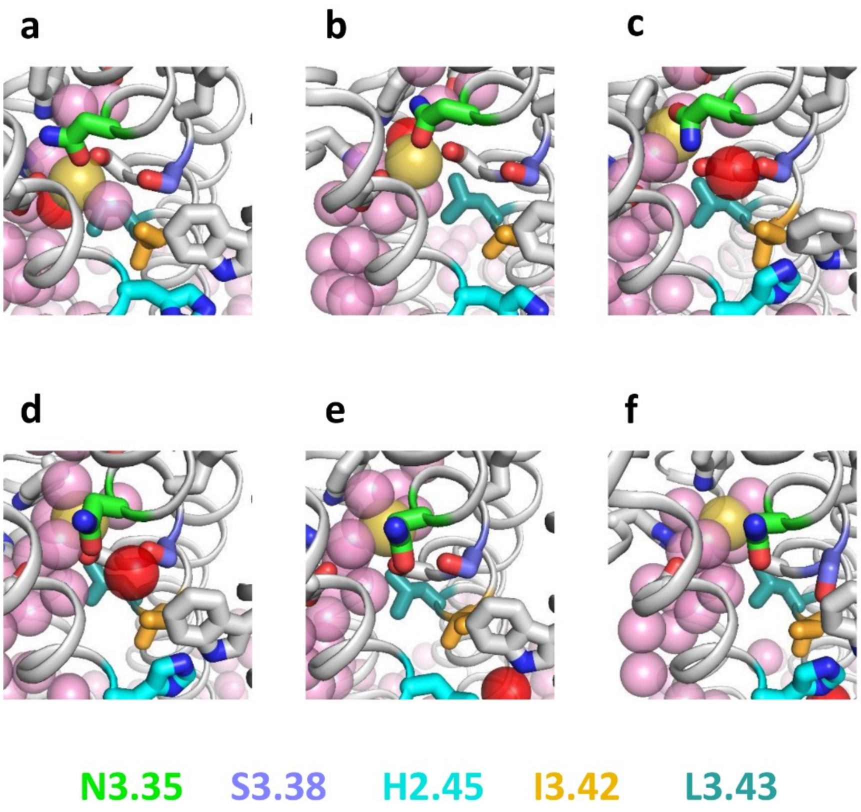

Supplement: Supplementary file 1 — Supplementary Information. [file 41598_2020_72766_MOESM1_ESM.pdf]
